# Can items derived from international literature be used in national quality of life instruments? A qualitative study conceptualising the EQ-HWB in China

**DOI:** 10.1186/s41687-024-00767-z

**Published:** 2024-08-05

**Authors:** Guangjie Zhang, Zhihao Yang, Nan Luo, Pei Wang, Jan Busschbach

**Affiliations:** 1 Department of Psychiatry, Section Medical Psychology, Erasmus MC, Rotterdam, The Netherlands; 2 Health Services Management Department, Guizhou Medical University, Guiyang, China; 3 Health Systems and Behavioural Sciences Domain, Saw Swee Hock School of Public Health, National University of Singapore, Singapore, Singapore; 4 School of Public Health, Fudan University, Shanghai, China

**Keywords:** Face validity, Semi-structured qualitative interview, Thematic analysis

## Abstract

**Introduction:**

The EQ Health and Wellbeing (EQ-HWB) is a new questionnaire for measuring quality of life (QoL) from a broad perspective. The items of the EQ-HWB were derived based on a ‘qualitative review’ of literature, which reported primarily on Western studies. It can be argued that the QoL is a cultural-related concept and therefore people from China have a different understanding of the QoL. This study aimed to explore whether Chinese citizens could understand the EQ-HWB’s candidate items and what they thought of those items. In doing so, we wanted to examine the face validity of the candidate items and explore if further cultural adaptation is necessary.

**Methods:**

This research was part of the E-QALY project, in which 36 candidate items were selected for the EQ-HWB from a 97-item pool. In China, three interviewers investigated the face validity of these EQ-HWB candidate items in semi-structured qualitative face-to-face interviews. Respondents were invited to report ‘problems’ with regard to the interpretation of the items and these problems were grouped into themes. We explored to what extent those themes related to specific cultural aspects in China. We also classified the rates of reported problems for each item into three groups: 1) less than 20%, 2) from 20–50%, and 3) over 50%.

**Results:**

For 17 items the rate of reported problems was less than 20%, 15 items fell into the second group (with 20 − 50%) and for 4 items the rate of problems reported was more than 50%. The thematic analysis revealed eight themes: ambiguous problems in the interpretation of 16 items; difficult to understand (11); contained a complex negative expression (10); examples used seemed inappropriate (7); misleading connotation in Chinese (2); long and complex (2); complex response options (1); and use of non-colloquial language (1).

**Discussion:**

Our research shows that EQ-HWB candidate items require careful examination to make them more comprehensible. Most of the reported problem themes were generic problems related to the items, and only a few face validity issues appeared to relate to specific cultural aspects in China, even though most of the items were based on Western studies. Our findings are reassuring for the instrument’s international application, especially in China.

## Introduction

There is an increasing demand to measure health-related quality of life (HRQoL) more broadly, often encompassing what is referred to as ‘well-being’ [1–3]. One reason given is that health interventions have an impact on more than just patients’ health [3, 4]. Another reason is that many health interventions, are not only for the benefit of patients themselves in terms of HRQoL, but also for the well-being of their carers [5, 6]. As a result, especially when making resource allocation decisions across sectors, e.g. healthcare and social care sectors, only considering HRQoL may not be sufficient, and well-being should be included as well [2, 7]. This is especially true when healthcare and social care are paid for from related budgets. For this reason, a generic preference-based instrument for capturing both health and well-being across healthcare and social care sectors and suitable for economic evaluation was warranted. The E-QALY project was initially inspired by the National Institute for Health and Care Excellence (NICE) [8]. Following on from this, the University of Sheffield [9] and the EuroQol group [10] cooperated in designing and generating one generic instrument (EQ-HWB™) for measuring broader quality of life (QoL) that cover both HRQoL and well-being across healthcare and social care sectors.

There were five stages of developing the EQ-HWB: (1) a literature review to identify potential domains; (2) item generation; (3) cognitive debriefing to test the face validity of candidate items; (4) psychometric analysis for candidate items and (5) the final item selection [11]. Particularly striking is that all references used for the qualitative review method for the determination of the domains of the EQ-HWB in the first stage were from Western articles [12]. It could be argued that these international articles were mostly based on studies that focused on Western countries and might not validly represent QoL at a national level, especially for non-Western countries. One risk incurred was that the items and domains used in the EQ-HWB could not represent an Eastern view of the QoL.

This international approach to developing the new EQ-HWB instrument was uncommon, contrasting with most instruments that started in a single country or language and then moved to translations [13]. In doing so, one was confronted with authors who claimed that the concepts of health-related quality of life (HRQoL) are in fact different between the Western countries and China [14, 15]. This view is supported by the idea that HRQoL is an individual’s subjective assessment of health, which is therefore impacted by any differences in cultural and societal background [16]. For example, a qualitative study found that ‘spiritual appearance’ was used by Chinese people to describe health, but this concept is hardly mentioned or discussed in the West [13]. In addition to cultural differences, translation issues should be considered as well. Most HRQoL instruments are designed by Western researchers, and one often assumes conceptual equivalence [17, 18] when translating the instruments into Chinese. But Yang et al. [19] reported that the ‘pain/discomfort’ and the ‘anxiety/depression’ domains of the EQ-5D-5L were poorly understood by the Chinese rural population. This was because ‘anxiety/depression’ is a Western term and was commonly used in Western society, but it is less frequently used in the Chinese language, especially in rural regions. For the reasons mentioned above, some Chinese researchers argued that the Western-designed HRQoL instruments may not be feasible in the Chinese population [14]. For example, it was found that the EQ-5D-5L had a higher ‘ceiling effect’ in China than in the Western countries [20]. For these reasons, researchers may worry that using an international qualitative review for extracting items to generate the EQ-HWB may not sufficiently represent the concept of QoL in the Chinese population. Note that these problems must be distinguished from items having generic interpretation problems, not specifically related to the Chinese context.

In this article we set out to explore the face validity of the items and domains used in the EQ-HWB in the view of the Chinese respondents, to see if the items deviate from the Eastern view of the QoL. Although Carlton et al. [11] reported face validity results for the item pool of all 97 candidate items in six countries, their study was focused on item selection of the generated 25 items of the EQ-HWB. Details about how the Chinese population responded to these candidate items were not reported by Carlton. It is essential to acknowledge that not all problems found were necessarily related to the Chinese context. It could well be that the problems found were generic, i.e. that the problems are likely to be found in any culture. An in-depth exploration of the problems in understanding the items, in the description itself, translation, or cultural mismatch has therefore been presented below.

## Methods

### Items

The research used the Chinese data collected as part of the above-mentioned study of six countries that collected similar data. The comparison between data sets was published by Carlton et al. [11]. Here we present an in-depth analysis of the Chinese data set.

A pool of 97 potential items was initially reduced to 36 items and later to 25 items. These 25 items were used in the long version of the EQ-HWB. The current study included the initial selection of 36 items. Given that our data was derived from the development stage, the wording of some of the 36 items were subtly different from the experimental version of the EQ-HWB.

### Interviewer training

Three postgraduate students at the School of Public Health of Fudan University in Shanghai were recruited and attended the interviewer training session. All three had experience with qualitative research and interviews prior to attending the training session. These interviewers were first introduced to the E-QALY project, after which the trainer introduced the interview process, and the trainees then conducted mock interviews with each other at the end of the training period. All interviewers were provided with training documents and videos, a protocol, and a topic guide. All processes were conducted in accordance with the original international protocol, which also includes references to supplementary material like the topic guide for face validity interviews [11].

### Participants

Respondents were recruited from the No.10 Hospital of Shanghai and the Zhongshan Hospital of Fudan University in Shanghai. Interviews were held from 26 July to 17 August 2018. We aimed to include patients with a wide variety of physical or psychological diseases, including frail old people and informal caregivers. Trained and qualified nurses were excluded as these professionals represent the professional perspective rather than the patient and caregiver perspectives that were the focus of the study. A quota was not set for socioeconomic variables, such as age, gender, education and ethnicity. Additional inclusion criteria were that: (i) respondents were older than 18 years of age, (ii) respondents consented to attending the interview, and (iii) respondents were able to fill out the questionnaires and provide comments on the items in a one-hour interview.

### Data collection

Data collection in Shanghai hospitals ensures that patients and informal caregivers from many different Chinese provinces are approachable, because high-quality medical resources are concentrated in large cities in China. The international protocol did not set specific sample size, but for pragmatic reasons the minimum was set at 30 respondents. This number had been suggested by the ISPOR guidelines for the cultural and translation process of questionnaires [21]. After respondents had consented and signed the informal consent, they were invited to attend face-to-face semi-structured interviews.

A total of 96 candidate items were reviewed in the face validity interviews. Considering the cognitive burden of respondents, each respondent only responded to a subset of a maximum of 50 items. After the respondents had read each item, interviewers asked the respondents how they interpreted each item, their ability to respond to the item and whether they could understand the items in terms of the topic guide instructions. The whole interview programme was recorded by an encrypted device. After the interviews, the interviewer listened to the interviews again. They did not transcribe the interview verbatim, but only recorded comments related to understandability and comprehensibility in an Excel file. After all the interviews had been completed, all comments were gathered and incorporated into one Excel file.

Of the 96 candidate items, 36 items were used for the ‘experimental version’ of the EQ-HWB and those 36 were used for the current study. These 36 items were divided into two groups. One group with 14 candidate items received none or only minor changes. Another group of 22 candidate items were modified or combined. For example, candidate items candidate items ‘I had trouble thinking clearly’ and ‘I found it hard to concentrate’ were combined into the item ‘I had trouble concentrating/thinking clearly’. Thus, the 14 items and the 22 combined/modified items together form the 25 items of the experimental version of the EQ-HWB.

### Data analysis

We used mixed methodologies including thematic analysis and numeric analysis to analyse data. Thematic analysis was used to explore whether the respondents understood the items, especially identifying which comments related to translation and cultural issues. The first author (GZ) of this article was not involved in the data collection interviews. GZ therefore first familiarised herself with the comments and problems of each item and then gave ‘themes’ for a group of comments and problems [4]. GZ then discussed preliminary results with JB, NL and ZY until consensus was reached. Subsequently, the number of comments and problems within these groups (themes) per item were counted and the themes per item were then ranked from the themes most mentioned to the themes least mentioned. In the ‘numeric analysis’, items were categorised into three groups based on the rate of the comments and problems reported. These three groups were: the rate of problems 1) was less than 20%, 2) fell between 20–50%, and 3) was more than 50%. The threshold of 50% can obviously be seen as arbitrary, but we would argue that if more than half of the respondents have comments on an item, it must be seen as problematic. This meant that we had an indication of how often comments and problems were given per item and, in general, which theme had the most noteworthy comments and problems over all items. Notably, we looked for themes specifically related to cultural and translational aspects in China.

## Results

### Participant characteristics

A total of 30 participants were recruited. The majority came from outpatient services, the others from inpatient services. Thirteen respondents were caregivers, ten were patients with physical problems, four were patients with mental problems, one was a patient with both physical and mental problems and the disease history of two respondents was not collected by interviewers. More details were presented in Table 1.

**Table 1:** Demographics of responders

Variable	Group	*N* = 30	%
Gender	Female	18	60.0
	Male	12	40.0
Age	< 20	2	6.7
	20–30	11	36.7
	30–40	5	16.7
	40–50	4	13.3
	50–60	5	16.7
	> 60	3	10.0
Education	Missing information	5	16.7
	Primary	2	6.7
	Junior High	3	10.0
	High	3	10.0
	College	4	13.3
	University	10	33.3
	Master’s degree & above	3	10.0
Experience	Carers	13	43.3
	Patients with physical problems	10	33.3
	Patients with mental problems	4	13.3
	Patients with both physical & mental problems	1	3.3
	Patients without disease categories registered	2	6.7
Ethnicity	Han (Majority)	24	80.0
	Tujia (Minority)	1	3.3
	Missing information	5	16.7

### Numeric analysis: problem frequency per item

The rate for reporting problems per item ranged from 0 to 79%, which was presented in Tables 2a and 2b. For 17 items the rate of the reported problems was less than 20%, for 15 items it was between 20 − 50% and four items had more than 50% problems. The themes for each item were expressed in the right-hand columns of Tables 2a and 2b.

**Table 2a:** Items used for in the experimental version of the EQ-HWB which were (almost) similar as the original items tested

Item	Domain	Original item	EQ-HWB experimental 25 version	Rate %	Themes of Respondents’ Comments
1	Relationship	I felt accepted by others	I felt accepted by others (felt like you were able to be yourself and that you belonged)	0	None
2	Feelings and emotions	I felt frustrated	I felt frustrated	0	None
3	Relationship	I felt unsupported	I felt unsupported by people	0	None
4	Activity	I could do the things I wanted to do	I could do the things I wanted to do	7	Difficult to answer the item with the given response options
5	Relationship	I felt lonely	I felt lonely	8	The expression of item is negative
6	Cognition	I had trouble remembering	I had trouble remembering	8	The expression of item is ambiguous
7	Physical sensation	I felt exhausted	I felt exhausted	8	The expression of item is negative
8	Autonomy	I felt unable to cope with my day-to-day life	I felt unable to cope with my day-to-day life	8	The expression of item is negative
9	Self-identify	I felt good about myself	I felt good about myself	9	The item is easy to mislead respondent
10	Physical sensation	I had discomfort, e.g. feeling sick, breathless, itching etc. (but not including pain)*	I had discomfort e.g. feeling sick, breathless, itching etc. (but not including pain)	15	The expression of item is ambiguous
11	Feelings and emotions	I felt anxious	I felt anxious	17	The expression of item is negative Difficult to understand the item
12	Feelings and emotions	I felt unsafe	I felt unsafe (fear of falling, abuse, or other physical harm)	25	The expression of item is ambiguous
13	Physical sensation	I had problems with my sleep	I had problems with my sleep	31	The expression of item is ambiguous
14	Feelings and emotions	I felt that I had nothing to look forward to	I felt I had nothing to look forward to	50	The expression of item is ambiguous The expression of item is negative Difficult to understand the item

*These items appear twice in the table, as they are combined with other items in different ways

**Table 2b:** Items which were modified or combined from original items, and used for the experimental version of the EQ-HWB version

Item	Domain	Original item	EQ-HWB experimental 25 version	Rate %	Comment
15	Activity	1 How well can you see (using your glasses or contact lenses if they are needed)?	How difficult was it for you to see? (using, for example, glasses or contact lenses if you usually use them)	50	Difficult to understand the item
		2 I had no difficulty seeing (using your glasses or contact lenses if they are needed)		50	Difficult to understand the item
16	Activity	1 Because of hearing and/or speech, how difficult did you find it to have a conversation?	How difficult was it for you to hear? (using, for example, hearing aids if you usually use them)	25	Difficult to understand the item The expression of item is ambiguous
		2 How well can you hear (using hearing aids if needed)?		50	Difficult to understand the item The expression of item is ambiguous
		3 I had no difficulty hearing (using hearing aids if needed)		58	Difficult to understand the item The expression of item is ambiguous The item is too long and complex to answer
17	Activity	1 I was able to get around inside my home with no difficulty	How difficult was it for you to get around inside and outside? (using, for example, walking stick, frame or wheelchair, if you usually use them)	25	The expression of item is ambiguous The expression of item is negative
		2 I was able to get around outside with no difficulty		17	Difficult to understand the item
18	Activity	1I had no difficulty with my day-to-day activities/ daily activities (e.g., working, shopping, travelling)	How difficult was it for you to do day-to-day activities (for example, working, shopping, housework)?	79	The example is inappropriate
19	Activity	1 Given the help I had/received my personal needs were met (e.g. being washed, going to the toilet, getting dressed, having food when I needed)	How difficult was it for you to wash, toilet, get dressed, eat or care for your appearance?	64	The example is inappropriate Difficult to understand the item
		I was able to look after myself (e.g. being washed, going to the toilet, getting dressed, having food when I needed)		31	The expression of item is ambiguous The example is inappropriate Difficult to answer the item with the given options
		2 I needed help with looking after myself (e.g. being washed, going to the toilet, getting dressed, having food when I needed)		31	The expression of item is ambiguous The example is inappropriate
		3 I was able to look after myself with no difficulty (e.g., washing, dressing, going to the toilet)		23	The expression of item is negative The item is too long and complex to answer
		4 I had no difficulty with self-care activities (e.g., washing, dressing, going to the toilet)		0	The example is inappropriate
20	Autonomy	1 I felt in control of my daily life	I felt I had no control over my day-to-day life (had the choice or do things or have things done for you as you liked and when you wanted)	38	Difficult to understand the item
		2 Which of the following statements best describes how much control you have over your daily life?		54	The item is easy to mislead respondent
21	Cognition	1 I had trouble thinking clearly	I had trouble concentrating/thinking clearly	8	The expression of item is ambiguous
		2 I found it hard to concentrate		8	Difficult to understand the item
22	Feelings and emotions	1 I felt sad	I felt sad or depressed	25	The expression of item is negative
		2 I felt depressed		8	The expression of the item is not colloquial
23	Physical sensations	1 How often do you experience pain *	I had physical pain?	15	The expression of item is ambiguous The expression of item is negative
24	Physical sensations	1 I had no pain	Please tick one box to describe your experience in the last 7 days: I had no physical pain I had mild physical pain I had moderate physical pain I had severe physical pain I had very severe physical pain	23	The expression of item is ambiguous
		2 How often do you experience pain *		15	The expression of item is ambiguous The expression of item is negative
25	Physical sensations	1 I had no discomfort e.g., feeling like throwing up, breathless, itching etc. (but not including pain)	Please tick one box to describe your experience in the last 7 days: I had no physical discomfort I had mild physical discomfort I had moderate physical discomfort I had severe physical discomfort I had very severe physical discomfort	46	The expression of item is ambiguous The example is inappropriate
		2 I had discomfort e.g., feeling sick, breathless, itching etc. (but not including pain)*		15	The example is inappropriate

*These items appear twice in the table, as they are combined with others in different ways

### Thematic analysis results

We generated eight themes in total. We ranked the frequencies of each theme from most to least. The respondents found the items to be ambiguous (16 times); difficult to understand (11 times); to have been expressed negatively (10 times); to have inappropriate examples (7 times); misleading (2 times); too long and complex to answer (2 times); difficult to answer using given response options (1 time); and the item was not expressed in colloquial language (1 time). Each theme was described below, including examples.

#### Ambiguous

The expression of the item could be ambiguous. It missed a clear scope or clear objective, for instance, implying that the way it was expressed allowed respondents to misinterpret the item. Examples were:

Item 14: ‘I felt that I had nothing to look forward to.’ *“Difficult to understand the question*,* as the item misses an object or scope.”*

Item 16 − 3: ‘I had no difficulty hearing (using hearing aids if needed).’ *“The sentence in the bracket makes this item ambiguous.”*

Item 10: ‘I had discomfort, e.g., feeling sick, breathless, itching, etc. (but not including pain).’ *“Here are many kinds of discomfort*,* and those listed in the questionnaire are also considered*,* and there are many other aspects*,* such as wearing clothes and shoes that are not suitable*,* will also be uncomfortable.”*

Item 16 − 2: How well can you hear (using hearing aids if needed)? *“Is it asking about hearing without assistance or after assistance?”*

#### Difficult to understand

This theme considered the comprehensibility of items, i.e. whether some words were difficult to understand. In some cases, the examples provided within the brackets had the opposite of the intended effect, thus confusing respondents.

Item 11; ‘I felt anxious’: *“What does ‘anxious’ mean?*“

Item 15 − 2: ‘I had no difficulty seeing (using your glasses or contact lenses if they are needed).’ *“The sentence in the brackets makes this item difficult to understand*.”

Items 19 − 1: ‘Given the help I had/received my personal needs were met (e.g., being washed, going to the toilet, getting dressed, having food when I needed)’. *“Healthy people don’t need help*,* and the question is not suited for healthy people to answer.”*

Item 15 − 1: ‘How well can you see (using your glasses or contact lenses if they are needed)?’ *“I wear glasses*,* so are you asking me how my vision is with glasses? I feel that the parentheses after the question are not easy to understand.”*

Item 16 − 3: ‘I had no difficulty hearing (using hearing aids if needed).’ *“The sentence in the bracket makes this item ambiguous.”*

#### Expressed negatively

This theme considered the emotional undertones of items. Some expressions may have triggered negative emotions in respondents. For example, ‘anxious’ and ‘nothing to look forward to’ induced respondents to think or imagine that unlucky or unhappy things would happen.

Item 14: ‘I felt that I had nothing to look forward to’. *“The question is too depressed*,* loss expectations.”*

Item 11: ‘I felt anxious.’ *“Bringing up such problems makes people feel stressful.”*

#### Inappropriate examples

The contents in brackets of some items did not explain the item clearly or the example was inappropriate.

Item 19 − 1: ‘Given the help I had/received my personal needs were met (e.g., being washed, going to the toilet, getting dressed, having food when I needed)’ *“The examples are inappropriate*,* and personal need also includes psychological need.”*

Item 18 − 1: ‘I had no difficulty with my day-to-day activities/ daily activities (e.g., working, shopping, traveling).’ *“Travel is not a daily activity.” “Working as a part of everyday life*,* it doesn’t work for retired seniors.”*

#### Misleading

The literal meaning of the word was not in line with the implicit meaning in Chinese. This theme focused on some words that had several connotations and some items that lacked usage contexts. Some respondents thus differed in their understanding of the item’s connotation.

Item 9: ‘I felt good about myself”. “*Felt good about oneself is a negative expression when translated into Chinese and has a meaning of being overconfident and arrogant.”*

Item 20 − 2: ‘Which of the following statements best describes how much control you have over your daily life?’. “*This has also a negative*,* derogatory meaning in China*. “.

#### Too long and complex to answer

Some long and complex items increased the cognitive burden for respondents. This could make respondents reluctant to read items seriously.

Item 16 − 3: ‘I had no difficulty hearing (using hearing aids if needed).’ *“Item is long and complex.” “The question makes respondent feel uncomfortable and refuses to answer the question.”*

Item 19 − 3: ‘I was able to look after myself with no difficulty (e.g., washing, dressing, going to the toilet).’ *“The content of this item is too repetitive and takes up space. The options are long too*,* too repetitive*,* and take up space”*.

#### Difficult to answer given the response options

This theme focused on how well the items and response options matched.

Item 4: ‘I could do the things I wanted to do’ *“It is not appropriate to use the answer options given to answer.” (response option: none of the time*,* only occasionally*,* some of the time/ sometimes*,* often*,* and most or all of the time)*.

#### The item was not expressed in colloquial language

Item 22: ‘I felt depressed.’ *“Depressed is a word that is rarely used in real life*,* not colloquial enough to ask questions.”*

## Discussion

The purpose of this study was to explore the face validity of candidate items of the EQ-HWB in the Chinese population. Surprisingly, face validity did not appear to relate much to specific Chinese cultural aspects. Only for a few items was there an indication of a risk of ‘cultural adaptation’ problems. This low frequency of ‘cultural problems’ was a reassuring finding as it suggested that international literature, although more influenced by Western input, might not be as culturally ‘biased’ as one might expect in the case of developing a health and well-being scale.

The most frequently occurring themes were: ‘ambiguous’, ‘difficult to understand’, ‘expressed negatively’ and ‘inappropriate examples’. In addition, these themes included the four candidate items with a ‘problem rate’ higher than 50%. But these problems did not reveal univocal relationships with Chinese culture as they related to general problems that may occur in any culture or language. They mainly stemmed from inappropriate wording rather than conceptual issues.

Argentina also suggested some changes based on Argentina’s inappropriate wording [22]. For example, ‘needs’ was understood in different ways, i.e. not just for the intended hygiene or self-care aspects. The authors also provided examples of other items and proposed changes in the translation process. Consequently, the EQ-HWB developers identified many generic problems and most of the frequently mentioned problems were addressed by rephrasing the 25 items for the final EQ-HWB version. For example, for item 18 - ‘day-to-day activities’ - respondents thought ‘travel’ was not a daily activity, hence this example was deleted.

Evidently, it was difficult to fully disentangle which problems were culture-related and which problems were generic. Clearly, not all problems reported could be described as being dependent on Chinese culture. But three items (‘I felt good about myself’, ‘control of day-to-day life’ relating to the theme of ‘misleading connotation’) and (‘I felt depressed’ relating to the theme ‘non-colloquial’) appeared to pose cultural problems. When these items were put to the test in China they did not appear to establish concept and semantic equivalence with the original English items [18]. The translation of the item ‘I felt good about myself’ into Chinese had the underlying meaning of being arrogant and overconfident. Clearly, this was not the intended meaning of the item in a health and well-being instrument. Another problematic item in the Chinese context was the conceptual equivalence of the phrase ‘I felt depressed’. In the testing face validity of 96 candidate items, the translation of ‘depressed’ is ‘沮丧’. This translation may be problematic, because another word ‘郁闷’ would be more often used in Chinses daily language. Although the item ‘depression/anxiety’ in the EQ-5D-5L instrument was translated into ‘沮丧’ in simplify Chinese version, but the ‘depression’ is a ‘Western’ diagnostic entity [23]. While Care et al., reported that ‘I feel depressed’ possessed ‘venting one’s emotion’ and other language elements [24]. Therefore, ‘I feel depressed’ was not the same as the ‘depression’. Similarly, ‘how much can you control your daily life’ had the same conceptual equivalence problems. ‘Control’ is a word with a negative connotation in Chinese culture and it describes a hierarchical relationship, where the person higher in the hierarchy controls the person lower in the hierarchy. Moreover, control is not a word collocated with ‘daily life’. However, these problems appear to be linguistic rather than cultural problems. Given the view chosen in this study and the problems found in relation to the 36 items, on which the 25 items of experimental version of the EQ-HWB is based, scrutinising the items in general for inconsistencies, and looking again carefully at the translation of the items into Chinese constitute a worthwhile exercise.

In contrast, a surprising finding was that two of the three items had a comparatively low problem frequency. *“I felt good about myself’’* had a problem rate of 9% and for “*I felt depressed”* this was 8%. From this perspective, these items may not prove to be a major problem in the final Chinese EQ-HWB version. However, the rate for the item: *“Which of the following statements best describes how much control you have over your daily life?”* was 54%. This meant that only one of the 25 items where face validity was tested in the final EQ-HWB version appeared to pose a significant problem for the Chinese sample. These findings illustrated how complex and difficult it is to develop HRQoL and well-being items in general, irrespective of language or cultural features. When looking at the difficulties the Chinese respondents had with some items, the dilemma emerged that it was a fine balance between the necessity of cultural adaptations when deemed essential to facilitate understanding, and the need to maintain the generic nature of the instrument and to promote standardisation across countries as far as possible to facilitate international comparisons.

There are reports in literature that the WHOQOL-100 instrument development was in line with the results found in the present study. The general items of the WHOQOL-100 instrument were developed at various locations and the national-specific items were developed alongside the general items by addressing some important aspects that had been overlooked. However, the psychometric properties results found that the national-specific items performed no better than the general items [25]. Thus, although the investigators of the WHOQOL started with the idea that QoL was culturally related and although they were able to present cultural items that could be considered locally relevant, when bringing it all together, there was sufficient common ground to make an international questionnaire with valid local performance [25–27]. This is in line with our finding that most of the internationally generated items are well understood at a local level. Another commonality with the development of WHOQOL was that respondents preferred to answer brief and easy questions. Indeed, it could be observed from Tables 2a and 2b that the longer and more complex items received more remarks, indicating a negative relationship between item length and items’ understandability and readability. Long items may complicate respondents’ ability to find scope or objective, may introduce connotations and may contain complicated contextual introductions [28]. Notably there is a balance between long and short items. Short items without a context background raised the uncertainty and concerns of the items, while long items were required respondents possessed higher readability to answer the sentence [11].

One of the lessons learned from this study is that most of the comments made about the items refer to generic characteristics of the items, rather than aspects that must be understood as ‘typically Chinese’. This helps us understand the results of other investigations, for instance when Chinese respondents mention missing certain dimensions in Western questionnaires. This finding is interpreted in literature as if Western questionnaires are incomplete: *“The studies identified several health dimensions*,* such as “spirit (Shen)”*,* “body constitution” and “sleep”*,* which were highlighted in the Chinese literature and by Chinese lay participants*,* but have not been commonly covered in Western HRQoL measures such as EQ-5D.”* [12]. However, such remarks assume that Western respondents do not consider any additional dimensions to be relevant. This is highly unlikely [16, 19, 29]. What we need to know is whether different cultures have a different ordering of dimensions of HRQoL and not whether respondents can think of other things that might be important. Note that the EQ-5D and the EQ-HWB aim at scoring systems that are weighted by the national populations. Thus, Chinese citizens will ‘value’ the EQ-5D and EQ-HWB dimensions. This culturally sensitive weighting will be in line with the idea that the HRQoL outcome should be culturally valid. Needless to say, if an important ‘Chinese’ dimension is missed, the outcome may not be ideal. To bring this argument back to our present study: so far we have found few ‘typical Chinese difficulties’ in the interpretation of the results. We did find that Chinese respondents had difficulty understanding some items, but those difficulties seemed universal: they were complex items to understand regardless of who you asked [11]. This should worry the international developers of the EQ-HWB, not necessarily the Chinese developers of the EQ-HWB.

In summary, our findings, along with the literature, indicate that HRQoL and well-being instruments could be developed based on literature research, without moving into many cultural complications. When considering health and well-being there appears to be more that unites us internationally than divides us. Perhaps we like to think that we are all unique, in the sense of being a Frenchman, Russian, or Chinese, but essentially, we differ only in particular details. Indeed, many of the aspects of HRQoL have a logical ordering, which makes it difficult to see how cultural differences could influence this. For example, it is unthinkable that there are countries in which citizens prefer more pain to less pain or prefer two broken arms to one broken arm. Moreover, well-being has similar traits: is there a country where the inhabitants would prefer illness, unemployment, or being outcasts? To date, the evidence appears to suggest that we are very much alike, and only differ in the details.

### Limitation

The study was not without limitations. We used a convenience sampling approach in only two hospitals, which may limit the representativeness. For instance, patients and informal caregivers in the community (i.e. those not in the hospital) may be overlooked and therefore diversities are reduced. Furthermore, data saturation was not used as a stopping criterion for the sampling of respondents. Moreover, respondents with reading problems were excluded. Secondly, we could not achieve a full checklist of the COREQ guideline, for instance we did not have a follow-up with participants after the interviews to allow participants to give feedback on our findings. Although the topic guide directs the respondents in providing us with the problems they have in interpreting and responding to the items, the focus on problems prevents positive remarks from being made or being noted by the interviewers. This is a deviation from most qualitative work, which would allow for both negative and positive comments. Another limitation is that two themes only have one example for support the theme and some themes share overlapped examples.

## Conclusion

The internationally-derived items used in this study already have generic qualities when evaluated by Chinese respondents. Respondents are critical of the items in a generic sense rather than related to any specific Chinese aspects. It is a reassuring result for other internationally developed QoL questionnaires that results presented in international literature are mostly generic in relation to valid applications in different cultures. With respect to the EQ-HWB instrument employed in this paper, we found a number of items that were ambiguous and/or difficult to understand. This illustrates that developing items related to HRQoL and social well-being is complex, and our research suggests that the EQ-HWB requires further careful examination to make the items more comprehensible, for instance. Nevertheless, as the EQ-HWB is being developed with international ambitions in mind, this can be seen as a reassuring finding for its application in China. Moreover, this is an encouraging result for other internationally developed questionnaires.

## Data Availability

The dataset was shared on the Easy Dans repository, and the persistent identifier: 10.17026/dans-zyw-x44c.
